# Readiness for stoma reversal in patients with a diverting stoma: a concept analysis

**DOI:** 10.3389/fonc.2026.1958956

**Published:** 2026-10-30

**Authors:** Xian Zhou, Wen Xu, Yu Zeng, Sixuan Han, Yanyan Hong

**Affiliations:** 1 School of Nursing, Nanjing University of Chinese Medicine, Nanjing, China; 2 Department of General Surgery, Nanjing Hospital of Chinese Medicine Affiliated to Nanjing University of Chinese Medicine, Nanjing, China; 3 Department of Nursing, Nanjing Hospital of Chinese Medicine Affiliated to Nanjing University of Chinese Medicine, Nanjing, China

**Keywords:** concept analysis, diverting stoma, readiness for stoma reversal, rectal cancer, stoma reversal

## Abstract

**Background:**

Readiness for stoma reversal is an important concept for patients with a diverting stoma during the transition from “living with a stoma” to “resumption of transanal defecation and adaptation to postoperative bowel function.” However, its conceptual meaning remains unclear, limiting the development of related research and clinical practice. Therefore, it is necessary to clearly define the concept of readiness for stoma reversal in patients with a diverting stoma.

**Objective:**

To clarify the concept of readiness for stoma reversal in patients with a diverting stoma using Rodgers’ evolutionary concept analysis.

**Methods:**

Relevant studies on readiness for stoma reversal in patients with a diverting stoma were systematically searched in PubMed, Web of Science, ScienceDirect, CINAHL, the Cochrane Library, Embase, CNKI, Wanfang Data, and VIP Database. Rodgers’ evolutionary concept analysis was used to synthesize and analyze the included studies. The conceptual attributes, antecedents, consequences, and related concepts of readiness for stoma reversal in patients with a diverting stoma were extracted and summarized.

**Results:**

The core attributes of readiness for stoma reversal in patients with a diverting stoma include pre-reversal assessment, physical and functional readiness, information and knowledge readiness, and psychological readiness. The main antecedents included rectal cancer control and anastomotic healing, systematic health education and health literacy, socioeconomic conditions and support systems, and multidisciplinary assessment and collaboration. For patients, adequate readiness may facilitate bowel function recovery, enhance engagement in rehabilitation and self-management, reduce anxiety and fear, lower the risk of postoperative complications, and improve quality of life. For healthcare systems, it may help shorten hospital stays, reduce readmissions and other unplanned healthcare use, and lower related medical costs.

**Conclusion:**

Readiness for stoma reversal is a multidimensional and dynamic state that develops before reversal in patients with a diverting stoma. It reflects the patient’s ability to cope with stoma reversal, bowel function recovery after reversal, and related lifestyle changes. Clarifying the attributes, antecedents, and consequences of this concept may support the development of assessment tools for readiness for stoma reversal. It may also provide a basis for designing individualized and continuous nursing interventions.

## 1 Introduction

Colorectal cancer (CRC) is one of the most common malignancies of the digestive system. In 2022, there were an estimated 1.926 million new cases and 904,000 deaths worldwide. CRC was the third most commonly diagnosed cancer and the second leading cause of cancer-related death globally, posing a major threat to public health. In recent years, the global burden of CRC has continued to increase owing to population aging, changes in dietary patterns, and lifestyle changes. By anatomical site, rectal cancer accounts for approximately 38.1% of all CRC cases, and middle and low rectal cancers are also relatively common (1).

Enterostomy is an important surgical procedure for treating colorectal cancer, saving patients’ lives, and prolonging survival. With advances in total mesorectal excision, low anastomosis techniques, and perioperative management, an increasing number of patients with mid- and low rectal cancer can preserve anal function (2). To reduce the risk of anastomotic leakage and related severe complications, a diverting stoma is commonly constructed after sphincter-preserving surgery for low rectal cancer. This allows fecal flow to be temporarily diverted through the stoma, reducing stimulation and contamination of the anastomosis by intestinal contents and thereby creating favorable conditions for anastomotic healing (3). Existing studies have shown that a diverting stoma can improve clinical outcomes in patients who develop anastomotic leakage after rectal cancer surgery (4). However, a diverting stoma is not the endpoint of treatment. Stoma reversal represents a key stage in the patient’s transition from “living with a stoma” to “resumption of transanal defecation and adaptation to postoperative bowel function.”

At present, most patients with a diverting stoma undergo elective stoma reversal after anastomotic healing has occurred and when oncological treatment and general condition allow. In clinical practice, reversal is commonly performed approximately 3 months after the initial surgery (5, 6). Nevertheless, patients remain at risk of postoperative complications and readmission following stoma reversal. A large retrospective cohort study involving 6,160 patients reported a 30-day readmission rate of 5.0% following ileostomy reversal (7). Furthermore, restoration of intestinal continuity does not necessarily indicate recovery of normal bowel function. Following sphincter-preserving rectal cancer surgery, patients may experience a range of bowel dysfunction symptoms, including increased stool frequency, fecal incontinence, and clustering of bowel movements, collectively referred to as low anterior resection syndrome (LARS) (8). The reported prevalence of LARS ranges from 60% to 90%, and symptoms may persist for years after surgery (9). A systematic review and meta-analysis further reported a pooled incidence of major LARS of 44% among patients assessed one year after sphincter-preserving surgery (10). These bowel dysfunctions can substantially affect patients’ quality of life, emotional well-being, and social functioning. Furthermore, a qualitative systematic review revealed that patients often experience uncertainty regarding postoperative bowel function before stoma reversal, while bowel problems after reversal may lead to psychological distress, feelings of shame and stigma, and difficulties adapting to daily and social life (11).

These findings highlight that stoma reversal involves not only the restoration of intestinal continuity but also adaptation to changes in bowel function and daily life. Whether patients are adequately prepared before stoma reversal may influence their ability to cope with postoperative recovery, recognize and respond to complications, and engage in effective self-management. However, the concept of readiness for stoma reversal in patients with a diverting stoma has not been clearly and consistently defined in the existing literature. This conceptual ambiguity limits the development of standardized assessment tools, the implementation of targeted nursing interventions, and the translation of research findings into clinical practice. Therefore, this study employs Rodgers’ evolutionary concept analysis to clarify the meaning of readiness for stoma reversal in patients with a diverting stoma, identify its defining attributes, antecedents, and consequences, and distinguish it from related concepts, thereby providing a theoretical foundation for future research and the development of relevant assessment tools.

## 2 Methods

### 2.1 Literature search strategy

A search strategy combining subject headings and free-text terms was used, with the search fields limited to titles, abstracts, and keywords. Search terms were organized around three core concepts: temporary or diverting stoma, stoma closure or reversal, and readiness or preparation. The English search terms included “temporary enterostom*/temporary ileostom*/temporary colostom*/protective enterostom*/protective ostom*/protective ileostom*/diverting ostom*/defunctioning ostom*/defunctioning stoma*,” “stoma closure/stoma reversal/closure of stoma/colostomy reversal,” and “readiness/readiness for stoma closure/preparation for stoma closure.” Equivalent Chinese terms were adapted for Chinese databases. PubMed, Web of Science, ScienceDirect, CINAHL, the Cochrane Library, Embase, CNKI, Wanfang Data, and VIP Database were searched. The reference lists of the included studies were also screened as a supplementary source. The search period was from database inception to June 30, 2026. The search strategy was structured using Boolean operators to broaden the scope. **Figure 1** illustrates the PubMed search strategy, and the **Supplementary Material** provides detailed search strategies for the remaining databases.

**Figure 1:**
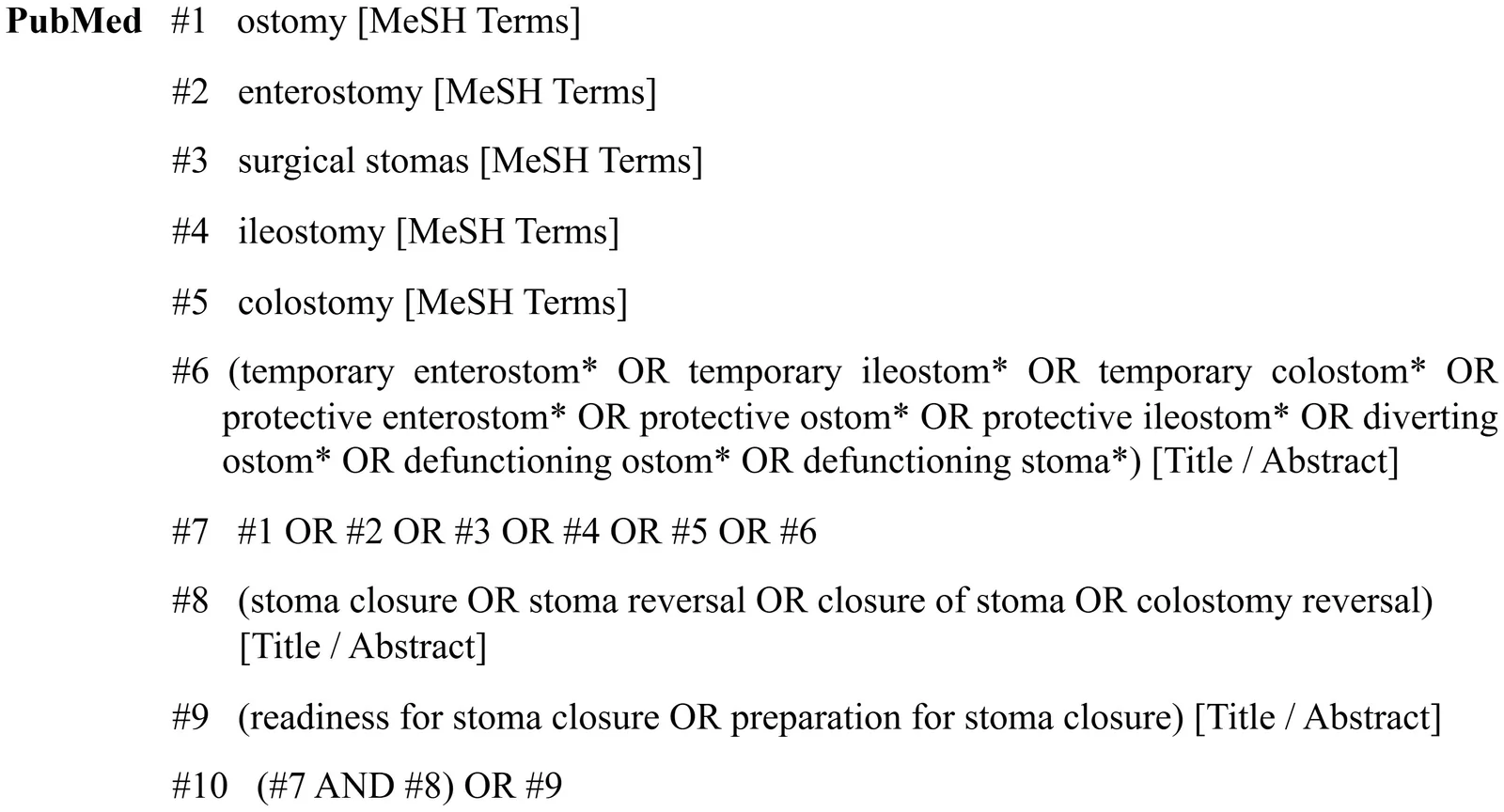
Search strategy used in PubMed.

### 2.2 Inclusion and exclusion criteria

The inclusion criteria were as follows: (1) the study population consisted of patients with a diverting stoma; (2) the study content involved at least one of the following aspects of readiness for stoma reversal in patients with a diverting stoma: conceptual meaning, related terms, antecedents, consequences, or cases; and (3) the publication language was Chinese or English. The exclusion criteria were as follows: (1) conference papers, abstracts, reviews, or studies that did not provide complete research information; (2) duplicate publications.

Literature screening was independently conducted by two researchers according to the inclusion and exclusion criteria. Any disagreement was resolved through discussion with a third trained researcher. The database searches identified 2,228 records. After removing 630 duplicates, 1,598 records underwent title and abstract screening, of which 1,394 were excluded. The full texts of 204 potentially eligible articles were assessed, and 165 were excluded for specified reasons. Ultimately, 39 articles were included in the concept analysis, comprising 27 English-language and 12 Chinese-language articles. The number of included studies met the requirements of Rodgers’ evolutionary concept analysis. According to the PRISMA flow diagram, the screening process is shown in **Figure 2**.

**Figure 2:**
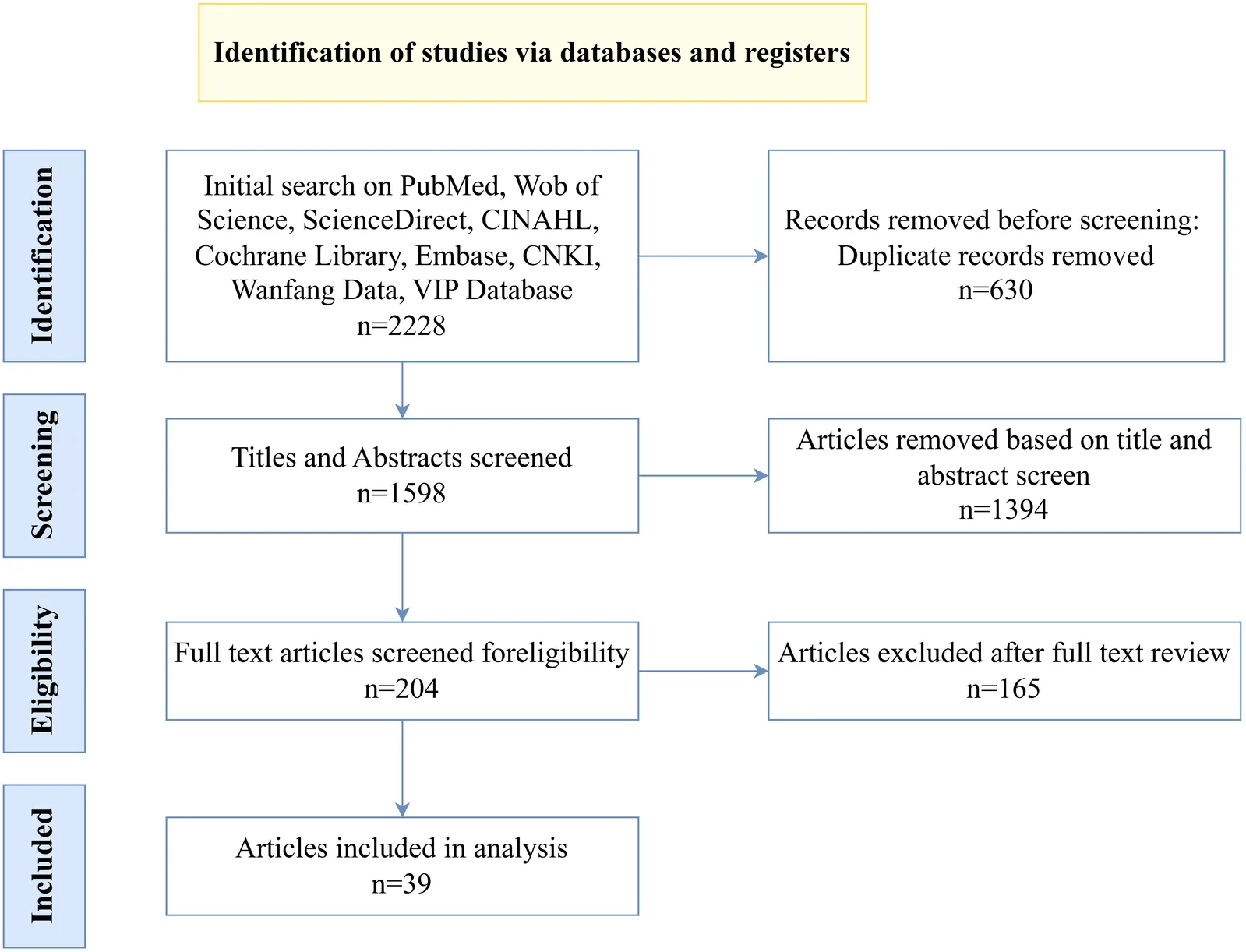
Prisma flow diagram of the study selection process.

### 2.3 Analysis process

This study analyzed the included literature using Rodgers’ evolutionary concept analysis. This method assumes that concepts evolve over time and across contexts. Its purpose is not to produce a fixed and unchanging definition, but to examine how a concept is used in a specific context and to reveal its current meaning, structure, and boundaries. Therefore, this study systematically analyzed the relevant literature to identify and summarize the surrogate terms, related concepts, antecedents, attributes, examples, and consequences of readiness for stoma reversal in patients with a diverting stoma. This process aimed to further clarify the meaning and scope of the concept (12).

Data extraction was independently completed by two researchers. Any disagreement was resolved through discussion with a third researcher until consensus was reached. Before formal data extraction, the two researchers randomly selected three included studies for preliminary extraction. The extraction results were then compared and discussed to ensure a consistent understanding of the extracted content. Based on this process, the data extraction form was revised, and the final extraction framework was developed. Subsequently, the two researchers independently extracted data using the revised data extraction form. This study was conducted according to the three phases of Rodgers’ evolutionary concept analysis (12). Initial phase: The concept to be analyzed and its research significance were identified. The disciplinary, clinical, and temporal contexts of the concept were clarified. The inclusion and exclusion criteria were developed, and the analytical materials were collected and screened. Core analysis phase: The included literature was repeatedly compared and synthesized. Surrogate terms and related concepts were identified, and the antecedents, attributes, examples, and consequences of the concept were extracted. The use of the concept across different studies and contexts was also analyzed. Further analysis phase: The results of the concept analysis were integrated to identify unresolved issues and propose hypotheses that could be tested in future studies. Directions for further conceptual development and clinical application were also clarified.

## 3 Results

### 3.1 Conceptual evolution

The Collins English Dictionary defines readiness as a state in which an individual is “willing to do something” or is “prepared” for a future event. Its synonyms include willingness, inclination, eagerness, and keenness, emphasizing an individual’s willingness, tendency, and state of preparation before taking action (13). In the fields of medicine and nursing, this concept is often understood in the context of changes in health status, lifestyle, or care settings. It is used to describe a comprehensive state of preparedness that individuals develop before disease treatment, functional recovery, or care transition. Fleury (14) suggested that individual readiness for change includes reassessing one’s lifestyle, identifying barriers in the change process, and developing a commitment to change. Fowler (15) further pointed out that nursing interventions should be delivered at the time when patients are most likely to succeed. Therefore, identifying whether patients are in an appropriate state of readiness is an important component of nursing practice. Thus, readiness does not simply refer to whether an individual is able to undergo a medical procedure. Rather, it emphasizes the individual’s cognitive, psychological, behavioral, and resource-related preparation for an upcoming change. In 2025, some researchers (16) defined readiness for stoma reversal as a state in which patients have good physical condition, psychological readiness, knowledge readiness, and a sound social support system to cope with the problems and needs encountered after stoma reversal. This definition emphasizes the importance of physical readiness, psychological readiness, knowledge readiness, and support system readiness in the process of stoma reversal.

Taken together, the above literature indicates that readiness for stoma reversal not only reflects the core meaning of readiness as a state of preparation before transition, but also has specialty-specific features in the context of stoma reversal. It includes not only whether patients meet the medical criteria for reversal surgery, but also whether they have the comprehensive ability to cope with postoperative bowel symptoms, reconstruction of defecatory function, risks of complications, lifestyle adjustment, and psychosocial adaptation.

### 3.2 Attributes

Defining attributes refer to the key characteristics, elements, or components of a concept. They help achieve a more accurate and in-depth understanding of the concept and avoid confusion with other similar concepts (17). Through literature reading and analysis, the following attributes related to readiness for stoma reversal were extracted: pre-reversal assessment, physical and functional readiness, information and knowledge readiness, and psychological readiness. The defining attributes of readiness for stoma reversal in patients with a diverting stoma are described from the following four aspects.

#### 3.2.1 Pre-reversal assessment

Pre-reversal assessment is an important basis for determining whether patients are eligible for stoma reversal. Before reversal, patients with a diverting stoma should undergo systematic assessment to clarify the control of the primary disease, the status of anastomotic healing, the conditions for restoring intestinal continuity, and pelvic floor function. A detailed and standardized preoperative assessment is not only a prerequisite for the successful implementation of stoma reversal, but also an important step in identifying the risk of postoperative complications and determining the patient’s level of readiness for reversal.

The Chinese Expert Consensus on Diverting Stoma in Surgery for Mid- and Low Rectal Cancer (2022 edition) (6) clearly states that patients with a diverting stoma should undergo rectal digital examination, MRI, CT, and pelvic floor function tests before reversal to fully assess anastomotic healing and the presence of local tumor recurrence or metastasis. Through these examinations, healthcare professionals can further identify factors that may affect the safety of reversal, such as anastomotic leakage, anastomotic stenosis, local recurrence, metastasis, or pelvic floor dysfunction. These findings provide a basis for selecting the timing of reversal and implementing perioperative nursing interventions (18, 19).

#### 3.2.2 Physical and functional readiness

Physical and functional readiness encompasses not only the medical conditions required for stoma reversal but also the functional preparation needed for postoperative recovery. As patients transition from living with a stoma to the resumption of transanal defecation, they must adapt to changes in bowel function after reversal.

In terms of functional readiness, patients need to prepare for changes in the route of defecation and the reactivation of anorectal function after reversal (16). LARS is a common bowel dysfunction after stoma reversal in patients with rectal cancer (20). It mainly manifests as increased stool frequency, urgency, clustering of bowel movements, a sense of incomplete evacuation, and varying degrees of fecal incontinence. During the period of stoma retention, the distal bowel and anal sphincter lack fecal stimulation and regular defecatory activity for a prolonged time. This may lead to disuse-related functional decline and increase the risk of difficulty adapting to bowel function after reversal (21).

Therefore, when the patient’s condition allows, regular pelvic floor muscle training and anal function exercises should be performed before stoma reversal. These exercises can strengthen pelvic floor muscle function, maintain the activity of the pelvic floor muscles and anal sphincter, and improve defecatory control and coordination. This may lay a foundation for transanal defecation and bowel function recovery after reversal (22).

#### 3.2.3 Information and knowledge readiness

Information and knowledge readiness is an important attribute of readiness for stoma reversal in patients with a diverting stoma. Before stoma reversal, patients should understand not only the purpose and process of the reversal procedure, preoperative examinations, and related precautions, but also the possible changes in bowel function and the risks of related complications after reversal (23). Importantly, patients need to recognize that stoma reversal does not necessarily lead to the immediate restoration of normal bowel function and that postoperative bowel dysfunction, including LARS, may occur. Adequate information and knowledge readiness is reflected in patients’ understanding of the transition from fecal diversion through a stoma to the resumption of transanal defecation. Patients should acquire relevant knowledge about dietary adjustment, pelvic floor function training, identification of abnormal symptoms, follow-up visits, and timely medical consultation (11, 24). Such knowledge may help patients anticipate and respond to challenges associated with stoma reversal and postoperative recovery.

#### 3.2.4 Psychological readiness

During the period of living with a diverting stoma, patients often reduce outdoor activities or even avoid social interaction because of difficulties in stoma care, concerns about odor or leakage, and fear of being viewed differently by others. These experiences may lead to negative emotions such as anxiety, depression, and low self-esteem (25). Before planned stoma reversal, patients may also experience fear, confusion, and uncertainty because of limited understanding of the reversal procedure, postoperative changes in defecatory function, and risks of complications (26, 27). Therefore, psychological readiness is an important attribute of readiness for stoma reversal. Psychological readiness is reflected in patients’ emotional acceptance of stoma reversal, their ability to cope with uncertainty and potential postoperative bowel dysfunction, and their confidence in adapting to subsequent changes in daily life.

### 3.3 Antecedents

#### 3.3.1 Rectal cancer control and anastomotic healing

Rectal cancer control and anastomotic healing are essential medical prerequisites for preparing for stoma reversal in patients with a diverting stoma. A diverting stoma is constructed to reduce the risk of severe complications such as anastomotic leakage after sphincter-preserving surgery for mid- and low rectal cancer. Therefore, disease stability and satisfactory anastomotic healing provide an important clinical foundation for subsequent reversal preparation (28). Before reversal, patients’ vital signs and general condition should be relatively stable. Stoma-related complications, such as peristomal dermatitis and parastomal hernia, should be fully assessed and effectively controlled. Active infection and obvious intestinal inflammation that may affect subsequent reversal assessment and recovery should also be excluded (29). When the original indication for stoma construction has largely resolved, there is no obvious infection in the abdominal incision or abdominal cavity, the distal bowel is patent without obvious stenosis, and no contraindications to stoma reversal are present, the feasibility of reversal surgery can be further assessed (30). However, educational, psychological, and rehabilitation preparation may begin before all medical criteria for reversal are fulfilled, according to the patient’s clinical condition.

#### 3.3.2 Systematic health education and health literacy

Systematic health education and rehabilitation guidance are important antecedents that facilitate the development of readiness for stoma reversal in patients with a diverting stoma. Healthcare professionals should provide continuous, staged, and individualized education throughout the pre-reversal period to address patients’ evolving preparation needs. Educational content should cover the purpose and feasibility of reversal, preoperative preparation, surgical risks, stoma-related complications, postoperative bowel dysfunction, symptom management, and follow-up care (11, 31, 32). In particular, patients may equate stoma reversal with the restoration of normal defecatory function and underestimate the risk of LARS and its potential impact on quality of life, social participation, and daily functioning (20). Therefore, pre-reversal education should help patients develop realistic expectations regarding postoperative recovery and equip them with strategies for managing potential bowel dysfunction. Furthermore, health literacy may also influence patients’ ability to access, understand, and apply reversal-related information. Patients with limited health literacy may encounter difficulties in understanding health information and implementing self-management strategies. Accordingly, healthcare professionals should tailor educational content and communication approaches to patients’ health literacy levels to facilitate informed decision-making and adequate preparation for stoma reversal.

#### 3.3.3 Socioeconomic conditions and support systems

Patients’ socioeconomic conditions may influence the development of readiness for stoma reversal by shaping access to healthcare services and caregiving resources. A large cohort study by Kuryba et al. found that patients with rectal cancer from more socioeconomically deprived backgrounds were less likely to undergo stoma reversal, suggesting potential disparities in healthcare access (33). A qualitative study further identified employment interruptions due to caregiving responsibilities, financial burdens, travel barriers, and limited access to health insurance information, as well as gaps in caregiving knowledge and skills (34). These challenges may restrict patients’ and families’ access to continuous care and support during pre-reversal preparation.

Family and social support may further facilitate patients’ engagement in pre-reversal preparation. Family members and primary caregivers can provide assistance by accompanying patients to medical appointments, learning disease-related knowledge together, supporting rehabilitation exercises, and offering emotional support. However, caregivers may also require professional guidance and emotional support to address caregiving challenges and adapt to their roles (34). In addition, specialized stoma clinics, community healthcare services, peer support, and remote follow-up can provide ongoing professional and social support for patients and their caregivers (35).

#### 3.3.4 Participation in multidisciplinary assessment

Participation in multidisciplinary assessment is an important external condition that supports the development of readiness for stoma reversal in patients with a diverting stoma. Preparation for stoma reversal involves interrelated medical, nutritional, functional, psychological, and self-management needs that cannot always be adequately addressed by a single discipline. Therefore, a single discipline is unlikely to fully meet patients’ complex preparation needs. Multidisciplinary collaboration enables healthcare professionals to integrate information from different specialties, identify unmet preparation needs, and develop coordinated care plans (36).

After the construction of a diverting stoma, continuous, collaborative, and individualized management should be provided by colorectal surgery staff, oncology staff, dietitians, rehabilitation therapists, and specialist nurses. Colorectal surgery staff should focus on stoma care, prevention of stoma-related complications, and follow-up assessment. Oncology staff should develop adjuvant treatment plans according to the patient’s condition and monitor treatment-related adverse reactions and nutritional status. Dietitians should provide targeted interventions for malnutrition, anemia, weight loss, and related problems (37). Rehabilitation therapists should guide functional training according to the needs of pelvic floor and defecatory function recovery (38). Specialist nurses should provide health education and emotional support related to stoma reversal (39). Through collaborative assessment, information sharing, and coordinated interventions, the multidisciplinary team can identify gaps in readiness, adjust care strategies according to patients’ changing needs, and support the progressive development of readiness for stoma reversal.

### 3.4 Consequences

Consequences refer to the specific outcomes resulting from a concept. These outcomes help reveal the social context in which the concept is applied (17). Readiness for stoma reversal may have dual effects on both patients and healthcare providers.

#### 3.4.1 Patient outcomes

Adequate readiness for stoma reversal may contribute to positive outcomes in patients’ physical condition, pelvic floor function recovery, psychological adjustment, and quality of life. Sufficient preparation for reversal helps patients correctly understand their disease status and reversal-related risks before surgery. It can also enhance their awareness of monitoring bowel symptoms and stoma-related complications and improve their ability to identify related complications (40). In addition, adequate readiness can help patients improve their nutritional status and enhance engagement in pelvic floor muscle training and related rehabilitation exercises. These changes may lay the foundation for anorectal function recovery and bowel function recovery after stoma reversal. Psychologically, higher readiness for stoma reversal can help patients develop realistic postoperative expectations and recognize that stoma reversal does not mean immediate restoration of normal defecatory function. This may reduce their anxiety and fear related to the reversal procedure, changes in defecatory function, and disease recurrence (41). Through adequate knowledge preparation, psychological adjustment, and social support, patients can gradually develop positive coping strategies, enhance self-efficacy, and strengthen confidence in rehabilitation (42). Collectively, these potential benefits may facilitate adaptation to postoperative bowel function and contribute to improved quality of life.

#### 3.4.2 Healthcare system outcomes

A higher level of readiness for stoma reversal may have positive effects on the healthcare system. Adequate preoperative assessment, identification of complication risks, nutritional optimization, rehabilitation training, and continuity of care can help reduce surgical delays, postoperative complications, and unplanned healthcare utilization caused by insufficient preparation. Previous studies have shown that preoperative nursing for stoma reversal can shorten hospital stay, reduce the risk of surgical site infection, and decrease readmission or reoperation rates (43). Moreover, during the waiting period before reversal, patients with an ileostomy may still be readmitted because of dehydration, high-output stoma, stoma outlet obstruction, and skin problems, thereby increasing healthcare resource consumption (44). Thus, adequate readiness for reversal can help shift risk management to an earlier stage, shorten hospital stay, reduce the need for readmission and complication management, lower related medical expenditures, and promote the rational use of healthcare resources.

### 3.5 Related concepts

Readiness for stoma reversal and preoperative preparation for stoma reversal are two concepts that are often confused. Readiness for stoma reversal refers to the degree to which patients with a diverting stoma are comprehensively prepared in terms of physical condition, psychological status, knowledge, and rehabilitation behaviors before planned stoma reversal. It places greater emphasis on whether patients have the ability to cope with the reversal procedure and bowel function recovery after reversal. It reflects the patient’s own state and level of preparation. Preoperative preparation for stoma reversal refers to a series of assessments, guidance, and interventions implemented by healthcare professionals before stoma reversal to ensure surgical safety and promote postoperative recovery. It mainly includes preoperative examinations, disease assessment, bowel preparation, nutritional support, psychological care, health education, communication of complication risks, guidance on pelvic floor function training, and perioperative nursing arrangements (45, 46). The primary aim of preoperative preparation is to help patients meet the requirements for stoma reversal through standardized nursing care and medical interventions. It also seeks to reduce the effects of surgical stress, minimize perioperative risks, and promote postoperative recovery. Although the two concepts are closely related, they differ in their focus. Preoperative preparation for stoma reversal emphasizes what healthcare professionals do and represents a process of perioperative care and clinical intervention. Readiness for stoma reversal emphasizes whether the patient is ready and represents the patient’s overall state of preparedness.

### 3.6 Model case

A model case demonstrates the defining attributes, antecedents, and consequences of a concept, facilitating its understanding and identification (17).

Mr. Yu, a 68-year-old farmer, had a limited educational background. In December 2025, he underwent sphincter-preserving surgery for rectal cancer with the creation of a diverting stoma. After the surgery, a multidisciplinary team evaluated his clinical condition, nutritional status, stoma care ability, and psychological needs, and developed an individualized rehabilitation plan. During hospitalization and follow-up period, healthcare professionals provided continuous education on stoma care, complication prevention, pelvic floor muscle training, and stoma reversal. With professional guidance and family support, Mr. Yu gradually acquired the knowledge and skills needed for stoma management and postoperative rehabilitation. Before reversal, assessments confirmed stable disease, satisfactory anastomotic healing, and improved nutritional and physical status. Mr. Yu understood the reversal procedure, its potential risks, and anticipated postoperative bowel function changes. He was psychologically prepared and actively engaged in preoperative examinations and rehabilitation training. The multidisciplinary team therefore considered him adequately prepared for reversal, and the procedure was successfully performed. During the postoperative follow-up period, Mr. Yu continued dietary management and bowel function training, experienced no serious complications, and reported satisfactory bowel function recovery, reduced anxiety, and good quality of life. This case illustrates the physical and functional, information and knowledge, and psychological attributes of readiness for stoma reversal, together with its antecedents and potential positive consequences.

### 3.7 Borderline case

Mr. Zhao underwent sphincter-preserving surgery for rectal cancer with the creation of a diverting stoma in April 2025. Following surgery, he received multidisciplinary care and regularly attended specialist follow-up visits with his family, who participated in education on stoma care and reversal. He maintained a positive attitude toward reversal and paid attention to his nutritional needs. However, his engagement in pelvic floor muscle training was inconsistent because he frequently forgot to perform the exercises. After completing chemotherapy in December 2025, he underwent stoma reversal following comprehensive assessment. Postoperatively, he experienced frequent bowel movements and perianal irritation, which adversely affected his psychological well-being and quality of life. He subsequently recognized the importance of pelvic floor rehabilitation and began regular training to support bowel function recovery. In this borderline case, Mr. Zhao demonstrated adequate information and knowledge readiness and positive psychological readiness, and was medically eligible for reversal. However, his preoperative functional readiness remained incomplete. This case highlights the multidimensional nature of readiness for stoma reversal, illustrating that medical eligibility alone does not necessarily indicate comprehensive readiness across its physical and functional, informational, and psychological dimensions.

### 3.8 Contrary case

Ms. Dong underwent sphincter-preserving surgery for rectal cancer with the creation of a diverting stoma one year earlier. Following an inadequate response to her previous chemotherapy regimen, stoma reversal had been repeatedly postponed. She had recently started a new chemotherapy regimen, and the feasibility and timing of reversal had yet to be determined. She expressed considerable anxiety about whether reversal would be possible, had limited knowledge of the procedure and its preparations, and had not initiated pelvic floor muscle training, rehabilitation, or nutritional preparation. In this contrary case, Ms. Dong’s medical eligibility for reversal had not yet been established, while her physical and functional readiness, information and knowledge readiness and psychological readiness were insufficient.

### 3.9 Operational definition

Readiness for stoma reversal in patients with a diverting stoma refers to a dynamic, multidimensional state of preparedness for the reversal procedure and subsequent changes in bowel function, complication risks, and lifestyle. It encompasses pre-reversal assessment, physical and functional readiness, information and knowledge readiness, and psychological readiness. Adequate readiness may foster realistic postoperative expectations, active engagement in rehabilitation and self-management, psychological adjustment, functional recovery, improved quality of life, and reduced complication-related healthcare resource use.

## 4 Discussion

This study reviewed the literature and applied Rodgers’ evolutionary concept analysis to systematically clarify the meaning, antecedents, and consequences of readiness for stoma reversal among patients with a diverting stoma. It also highlighted the context-specific nature of the concept and developed a dynamic conceptual framework that reflects its evolving nature (**Figure 3**). This framework helps distinguish readiness for stoma reversal from related concepts, particularly preoperative preparation for stoma reversal. Unlike other readiness concepts, readiness for stoma reversal encompasses not only patients’ physical condition, psychological state, knowledge, and coping capacity but also distinct stoma-specific considerations (16). In particular, patients must transition from living with a stoma to transanal defecation and adapt to bowel function after reversal. Readiness for stoma reversal can therefore be understood as a multidimensional state that reflects how well patients are prepared to undergo the reversal procedure and manage postoperative functional recovery. Its four core attributes are pre-reversal assessment, physical and functional readiness, information and knowledge readiness, and psychological readiness. These attributes are interrelated and mutually influential, collectively reflecting patients’ overall preparedness for stoma reversal and subsequent bowel function recovery and adaptation.

**Figure 3:**
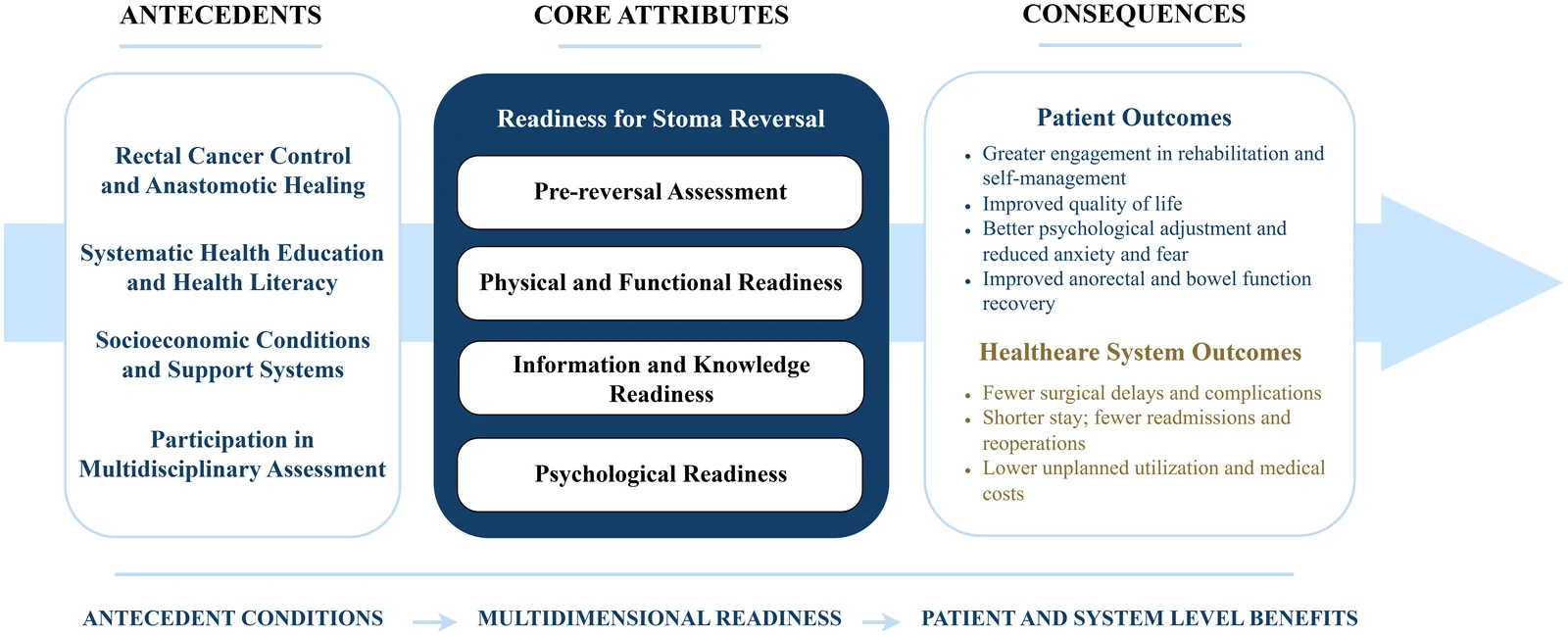
Conceptual framework of readiness for stoma reversal.

The findings suggest that readiness for stoma reversal may help patients develop realistic expectations regarding postoperative bowel function and strengthen their engagement in rehabilitation and self-management (42). These improvements may support bowel function recovery after reversal, alleviate anxiety and fear, reduce the risk of complications, and improve quality of life. At the healthcare service level, greater readiness may also reduce readmissions and other unplanned healthcare utilization, thereby limiting healthcare resource use (47). However, the relationships among the antecedents, attributes, and consequences are neither strictly unidirectional nor linear. Stoma-related complications, adverse effects of adjuvant therapy, changes in nutritional status, delays in reversal, and reduced family support may undermine patients’ readiness. In contrast, assessments at key stages and targeted interventions may address specific readiness gaps and help restore or enhance readiness. Readiness for stoma reversal should therefore be understood as both a multidimensional state at a given point before reversal and a dynamic process that evolves in response to disease progression, treatment experiences, available support, and changing rehabilitation needs. This perspective indicates that readiness cannot be adequately determined through a single assessment. Instead, it should be assessed longitudinally after stoma creation, during adjuvant therapy, when the reversal plan is established, and during preoperative preparation for reversal.

Readiness for stoma reversal may provide an important basis for individualized prehabilitation and continuity of care. The interval between stoma creation and reversal should not be regarded solely as a period of passive waiting for surgery. Instead, it may offer a valuable window for prehabilitation to improve patients’ physiological reserve, functional status, psychological adaptation, and self-management capacity (48). Future research could develop a condition-specific assessment instrument based on the core attributes identified in this study. Such an instrument could help identify gaps across different dimensions of readiness and guide stratified, targeted interventions. For example, patients with inadequate physical and functional readiness could receive enhanced nutritional support and rehabilitation training. Those with insufficient knowledge could receive structured education, whereas patients with unrealistic expectations of post-reversal function or substantial anxiety could benefit from expectation management and emotional support. This approach could shift clinical nursing from standardized, short-term preoperative education toward individualized and continuous support tailored to each patient’s readiness gaps. Readiness for stoma reversal is therefore not merely a theoretical concept used to describe a patient’s status. It may also have clinical decision-making value by providing a conceptual link among dynamic assessment, intervention selection, and outcome evaluation. This perspective offers a new theoretical basis and practical direction for optimizing stoma reversal care pathways.

## 5 Limitations

This concept analysis aimed to clarify and define readiness for stoma reversal among patients with a diverting stoma by reviewing literature from the major health and social sciences fields. However, this study has several limitations. First, readiness for stoma reversal was not explicitly defined in much of the existing literature. Although stoma care experts were consulted to refine the search terms and broaden the search scope, some relevant evidence may not have been captured. Second, the review was restricted to studies published in Chinese or English, which may limit the applicability of the findings across different linguistic and cultural contexts. Third, as this concept analysis was based on the literature, the identified attributes have not yet been validated against the experiences of patients undergoing stoma reversal. Future qualitative studies involving patients and healthcare professionals could further examine the relevance and comprehensiveness of these attributes. Finally, although an operational definition was proposed, specific measurement indicators and criteria for determining adequate readiness have not yet been established. Further research is needed to operationalize the identified attributes, develop appropriate assessment measures, and establish clinically meaningful criteria for evaluating readiness for stoma reversal.

## 6 Conclusion

Using Rodgers’ evolutionary concept analysis, this study systematically examined its antecedents, attributes, and consequences. Four core attributes were identified: pre-reversal assessment, physical and functional readiness, information and knowledge readiness, and psychological readiness. By clarifying its defining attributes, antecedents, and consequences, this concept analysis provides a theoretical foundation for developing and validating a condition-specific assessment instrument and guiding individualized nursing interventions during the pre-reversal period.

## Data Availability

The original contributions presented in the study are included in the article/**Supplementary Material**. Further inquiries can be directed to the corresponding author.
