# Supplementary material for: Readiness for stoma reversal in patients with a diverting stoma: a concept analysis
Source: Front Oncol. 2026 Oct 30;16:1958956. doi: 10.3389/fonc.2026.1958956 (PMC13759381; doi:10.3389/fonc.2026.1958956)
Supplement: Supplementary file 1 [file DataSheet1.docx]

Supplementary Material

# 1 Supplementary Figures

FIGURE 1 Search strategy used in PubMed.

FIGURE 2 PRISMA flow diagram of the study selection process.

FIGURE 3 Conceptual framework of readiness for stoma reversal.

**Supplementary Figure 4.** The figure legends are required to have the same font as the main text, 12 point normal Times New Roman, single spaced. Please use a single paragraph for each legend and prepare the figures keeping in mind the PDF layout.
