# Supplementary material for: Readiness for stoma reversal in patients with a diverting stoma: a concept analysis
Source: Front Oncol. 2026 Oct 30;16:1958956. doi: 10.3389/fonc.2026.1958956 (PMC13759381; doi:10.3389/fonc.2026.1958956)
Supplement: Supplementary file 2 [file DataSheet2.docx]

**1. Pubmed search algorithm**

#1	ostomy [MeSH Terms]

#2	enterostomy [MeSH Terms]

#3	surgical stomas [MeSH Terms]

#4	ileostomy [MeSH Terms]

#5	colostomy [MeSH Terms]

#6	(temporary enterostom* OR temporary ileostom* OR temporary colostom* OR protective enterostom* OR protective ostom* OR protective ileostom* OR diverting ostom* OR defunctioning ostom* OR defunctioning stoma*) [Title / Abstract]

#7	#1 OR #2 OR #3 OR #4 OR #5 OR #6

#8	(stoma closure OR stoma reversal OR closure of stoma OR colostomy reversal) [Title / Abstract]

#9	(readiness for stoma closure OR preparation for stoma closure) [Title / Abstract]

#10	(#7 AND #8) OR #9

**764 potential articles were retrieved.**

**2. Web of Science search algorithm**

#1	TS=(temporary enterostom* OR temporary ileostom* OR temporary colostom* OR protective enterostom* OR protective ostom* OR protective ileostom* OR diverting ostom* OR defunctioning ostom* OR defunctioning stoma*)

#2	TS=(stoma closure OR stoma reversal OR closure of stoma OR colostomy reversal)

#3	TS=(readiness for stoma closure OR preparation for stoma closure)

#4	(#1 AND #2) OR #3

**633 potential articles were retrieved.**

**3. ScienceDirect search algorithm**

Title, abstract, keywords: ((temporary enterostomy OR temporary ileostomy OR protective enterostomy OR protective ileostomy OR diverting ostomy OR defunctioning ostomy) AND (stoma closure OR stoma reversal)) OR (readiness for stoma closure)

**50 potential articles were retrieved.**

**4. CINAHL search algorithm**

#1	AB=(temporary enterostom* OR temporary ileostom* OR temporary colostom* OR protective enterostom* OR protective ostom* OR protective ileostom* OR diverting ostom* OR defunctioning ostom* OR defunctioning stoma*)

#2	AB=(stoma closure OR stoma reversal OR closure of stoma OR colostomy reversal)

#3	AB=(readiness for stoma closure OR preparation for stoma closure)

#4	(#1 AND #2) OR #3

**36 potential articles were retrieved.**

**5.** **Cochrane Library search algorithm**

#1	ostomy [mh]

#2	enterostomy [mh]

#3	surgical stomas [mh]

#4	ileostomy [mh]

#5	colostomy [mh]

#6	(temporary enterostom* OR temporary ileostom* OR temporary colostom* OR protective enterostom* OR protective ostom* OR protective ileostom* OR diverting ostom* OR defunctioning ostom* OR defunctioning stoma*) [ti,ab,kw]

#7	#1 OR #2 OR #3 OR #4 OR #5 OR #6

#8	(stoma closure OR stoma reversal OR closure of stoma OR colostomy reversal) [ti,ab,kw]

#9	(readiness for stoma closure OR preparation for stoma closure) [ti,ab,kw]

#10	(#7 AND #8) OR #9

**223 potential articles were retrieved.**

**6. Embase search algorithm**

#1	(temporary enterostom* OR temporary ileostom* OR temporary colostom* OR protective enterostom* OR protective ostom* OR protective ileostom* OR diverting ostom* OR defunctioning ostom* OR defunctioning stoma*) [ti,ab,kw]

#2	(stoma closure OR stoma reversal OR closure of stoma OR colostomy reversal) [ti,ab,kw]

#3	(readiness for stoma closure OR preparation for stoma closure) [ti,ab,kw]

#4	(#1 AND #2) OR #3

**265 potential articles were retrieved.**

**7.CNKI search algorithm**

(SU='临时性造口' + '保护性造口' +'预防性造口' + '暂时性造口') AND (SU='造口还纳' + '造口回纳') OR (SU='造口还纳准备度')

**67 potential articles were retrieved.**

**8.** **Wanfang Data search algorithm**

主题:("临时性造口" or "保护性造口" or "预防性造口" or "暂时性造口") and 主题:("造口还纳" or "造口回纳") or 主题:("造口还纳准备度")

**120 potential articles were retrieved.**

**9.** **VIP Database search algorithm**

M=(临时性造口 OR 保护性造口 OR 预防性造口 OR 暂时性造口) AND M=(造口还纳 OR 造口回纳) OR M=(造口还纳准备度)

**70 potential articles were retrieved.**
